# Enhancing Summer Tea Quality Through Integrated Shaking, Freezing, and Rolling Processing

**DOI:** 10.3390/foods14183159

**Published:** 2025-09-10

**Authors:** Changlian Wu, Huang Li, Qingxiu Lin, Zhong Wang, Chengzhe Zhou, Cheng Zhang, Yuqiong Guo

**Affiliations:** 1 Anxi College of Tea Science, Fujian Agriculture and Forestry University, Quanzhou 362400, China; cl3061095041@163.com (C.W.); zhongwang@fafu.edu.cn (Z.W.); chengzhechou@fafu.edu.cn (C.Z.); 2 College of Horticulture, Fujian Agriculture and Forestry University, Fuzhou 350002, China; 13984128485@163.com (H.L.); zc97422@163.com (C.Z.); 3 Fujian Qinghua Tea Industry Co., Ltd., Fuzhou 350015, China; qhcw0001@126.com; 4 Fujian Collaborative Innovation Center for Green Cultivation and Processing of Tea Tree in Universities, Fujian Agriculture and Forestry University, Quanzhou 362400, China

**Keywords:** summer tea, shaking, rolling, freezing, volatile compounds, process optimization

## Abstract

One of the main factors constraining the growth of the tea business is the low use rate of summer tea. To enhance the utilization rate and improve the quality of summer tea, this study innovatively integrated shaking, freezing, and rolling into the traditional processing methods of white tea. Processing parameters were optimized through single-factor experiments combined with an L_9_(3^4^) orthogonal experimental design. The quality of summer teas was systematically evaluated using sensory analysis, gas chromatography–mass spectrometry, and high-performance liquid chromatography. This study found that the optimal processing for summer tea was as follows: fresh leaves, room-temperature cold-air withering for 6.5 h, shaking at 10 rpm for 10 min, −20 °C freezing for 5 h, 25% strength rolling for 9 min, and drying at 75 °C for 2 h. The relative content of esterified catechins in summer tea produced by the optimal processing method was reduced by 14.62% compared with the control group. There were alterations in the content of amino acid components, with fresh and sweet amino acids increasing by 4.96% and 2.95%, respectively, and bitter amino acids reducing by 2.15%. Furthermore, γ-aminobutyric acid and L-theanine contents increased by 0.51% and 5.77%, respectively. Five characteristic volatile compounds were identified, namely, methyl salicylate, phenethyl formate, linalool, dimethyl sulfide, and isobutyraldehyde. The volatile profile was dominated by floral and fruity notes, except for dimethyl sulfide, which exhibited a distinct cooked corn-like aroma characteristic. This process was shown to improve the quality of summer tea. The results of this study provide a metabolite-level grounds for improving the quality of summer tea.

## 1. Introduction

Tea has gained global popularity as a beverage. It contains an abundance of polyphenols, amino acids, vitamins, and other active compounds, which have been shown to be beneficial to human health [1,2]. In China, tea is classified into three distinct categories based on the specific season during which it is harvested: spring tea, summer tea, and autumn tea. Traditional processing methods have a high utilization rate of spring tea and a low utilization rate of summer tea. However, China’s summer tea is abundant in fresh leaf resources, accounting for more than 60% of the annual total tea production [2]. Compared to spring tea, summer tea exhibits distinct chemical composition differences, containing lower levels of amino acids and other internal components while having higher concentrations of tea polyphenols [3]. These compositional variations result in increased bitterness and astringency along with reduced freshness in summer tea [4,5]. As a result, a substantial quantity of fresh tea leaves during the summer season remain unharvested, leading to low utilization rates [6].

Tea quality is determined by multiple factors, including the tea plant (*Camellia sinensis*) cultivar, plucking standards (e.g., bud-to-leaf ratio), and seasonal variations in fresh leaves, as well as processing techniques [7]. To enhance the utilization of summer tea resources, optimizing the processing of fresh tea leaves represents a viable approach for improving the quality of the tea. The process of shaking plays a pivotal role in shaping the distinctive aroma and flavor of oolong tea. During the shaking process, there is a continuous loss of water from the tea leaves; the leaves undergo mechanical damage; the activities of oxidase, hydrolase, and other enzymes are enhanced; and the substances contained in the tea leaves are altered. Research found that the shaking and standing process reduced the contents of catechins and proanthocyanidins in black tea, thus alleviating the bitterness and astringency of the tea broth [8]. Song et al. [9] found that, as the number of shaking treatments increased, the contents of tea polyphenols, the phenol-to-ammonia ratio, caffeine, and catechins in black tea initially rose and then declined, while the abundance of key odor-active compounds significantly increased after shaking. The shaking process promotes the oxidative degradation of fatty acids and carotenoids while modulating terpenoid biosynthesis, thereby enhancing the formation of floral and fruity aroma compounds [10,11]. Although oxidative reactions induced by freezing are generally considered detrimental in conventional food processing, they play an essential role in tea manufacturing. Studies have shown that freezing alters physicochemical properties by promoting browning and compositional breakdown but significantly improves organoleptic quality due to the browning effect [12]. Xia Yimin et al. [13] found that black tea subjected to combined freezing and shaking treatments exhibited a reduced tea polyphenol content, along with elevated levels of amino acids, theaflavins, and cis-3-hexenyl hexanoate. Tea rolling is an important step in the process of tea processing. Previous research findings indicated that the rolling time critically influences black tea quality parameters, with an extended rolling duration resulting in decreased catechin levels and the concurrent accumulation of volatile aromatic constituents [14]. Yang Shuya et al. [15] found that a prolonged rolling time significantly reduced the contents of major catechins—including (−)-epigallocatechin gallate, (−)-epicatechin, and (−)-epigallocatechin—in black tea production. Nie Zhanyi et al. [16] conducted a study in which they compared the effects of different freezing treatments on the quality of black tea. Their findings indicated that the aroma and flavor scores and total scores of tea samples obtained by freezing and then rolling were higher than those of tea samples that were first rolled and then frozen. These findings underscore the crucial role of processing methods in determining the final quality of tea. Although it has been demonstrated that shaking, freezing, and rolling could improve tea quality, systematic research on the synergistic mechanisms of these processes, their effects on the quality of summer tea, and the optimization parameters of the composite process is still lacking.

In this study, the optimal process parameters (including the shaking speed, freezing time, and rolling time) at different stages of the new summer tea process were evaluated using a combination of sensory evaluation, one-way analysis of variance (ANOVA), and orthogonal test analysis. Building upon these optimization results, we further examined the tea quality under the optimized summer tea processing conditions using quantitative descriptive analysis (QDA), high-performance liquid chromatography (HPLC), and gas chromatography–mass spectrometry (GC-MS). This study’s findings provide theoretical underpinnings and methodologies to enhance the quality of summer tea.

## 2. Materials and Methods

### 2.1. Chemicals and Reagents

All chemical reagents used in this study were of analytical grade. Disodium hydrogen phosphate dihydrate, oxalic acid, sodium carbonate, potassium dihydrogen phosphate, stannous chloride, sodium bicarbonate, alkaline lead acetate, ninhydrin, sodium chloride, 0.01 mol/L hydrochloric acid solution, 4.5 mol/L sulfuric acid solution, 95% ethanol, 75% methanol, ethyl acetate, and n-butanol were procured from Shanghai Yuanye Biotechnology Co., Ltd. Shanghai, China. A plant soluble sugar content test kit was obtained from Beijing Solebaum Technology Co., Ltd. Beijing, China. Additional reagents, including 4-tert-butylcyclohexanol and saturated sodium chloride (analytical grade), were purchased from Sinopharm Chemical Reagent Co., Ltd. Shanghai, China. Standard compounds with purity ≥ 98% were acquired from Chengman Lemaitian Pharmaceutical Science and Technology Co., Ltd. (Chengdu, China), including caffeine; (−)-epicatechin (EC), (−)-epigallocatechin (EGC), (−)-epigallocatechin gallate (EGCG), and (−)-epicatechin gallate (ECG); and Aspartic acid (Asp), Isoleucine (Iso), Threonine (Thr), Leucine (Leu), Serine (Ser), Tyrosine (Tyr), Glutamic acid (Glu), Phenylalanine (Phe), Glycine (Gly), Alanine (Ala), Histidine (His), Cysteine (Cys), Arginine (Arg), Valine (Val), Proline (Pro), γ-aminobutyric acid (GABA), Asparagine (Asp), Glutamine (Glu), L-theanine, Tryptophan (Try), and Lysine (Lys). Additional reagents, including sodium hydroxide, glacial acetic acid, methanol, sodium chloride, stannous chloride, acetonitrile, and tetrahydrofuran, were supplied by Shanghai Aladdin Biochemical Technology Co., Ltd. Shanghai, China.

### 2.2. Processing and Sample Preparation

The fresh leaf material used in this study consisted of Fuding Dahao tea, specifically one bud with two or three leaves, harvested during the summer season (August–September). The tea leaves were cultivated in the organic tea plantation of Fuding Jinding Tea Co. Ltd., located in Fuding City, Fujian Province, China (120°18′31″ E longitude, 27°24′15″ N latitude, 217 m altitude). For each experimental trial, a standardized quantity of 5 kg of fresh leaves was used.

Sample preparation for optimal process parameter investigation: The fresh leaf processing method that was implemented in the experiment comprised the following steps: withering, shaking, freezing, rolling, low-temperature drying, and gross tea. Withering was conducted under controlled conditions at 40 °C with 65% relative humidity, utilizing a 68 cm three-blade industrial fan with an airflow rate of 1400 r/min, continuing until the moisture content reached 52%. The shaking process was performed using a drum shaker at variable speeds ranging from 4 to 16 rpm. Subsequently, the samples were immediately packaged and subjected to freezing at −20 °C for durations between 2 and 10 h. Following freezing, the samples underwent crushing with cold-air cooling. The rolling process was conducted at 25% pressure (achieved by compressing the rolling machine cover to one-quarter of the drum height with a consistent tea leaf quantity) for 3 to 15 min. Final processing involved low-temperature drying at 75 °C for 2 h, reducing the moisture content to below 5% to obtain the finished tea samples. The experimental design incorporated three key factors, namely, shaking speed, freezing time, and rolling time, each examined at five treatment levels. Except for the individual process setting parameters, the rest of the processes were performed according to the parameters determined above. In the single-factor experiment for shaking speed, the shaking speed was set to 4 r/min, 7 r/min, 10 r/min, 13 r/min, and 16 r/min, denoted by Y1, Y2, Y3, Y4, and Y5, respectively; in the single-factor experiment for freezing time, the freezing time was set to 2 h, 4 h, 6 h, 8 h, and 10 h, denoted by L1, L2, L3, L4, and L5, respectively; in the single-factor experiment for the rolling time, the rolling time was set to 3 min, 6 min, 9 min, 12 min, and 15 min, denoted by R1, R2, R3, R4, and R5, respectively. The experimental parameters for the orthogonal array design are presented in Table S1. These parameters were systematically optimized based on the results obtained from preliminary single-factor experiments.

**Figure 1:**
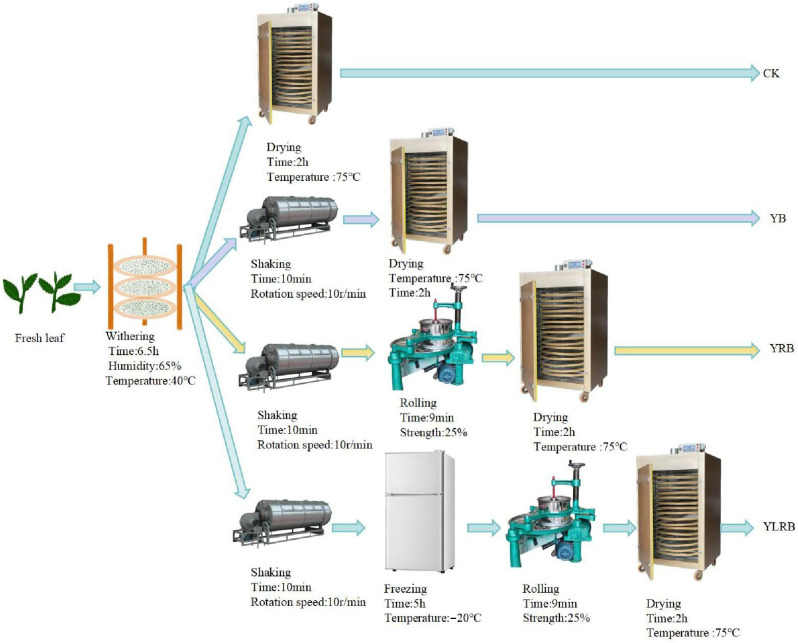
Process flow diagrams for four tea samples. **CK**: samples made with traditional white tea processing techniques; **YB**: sample made by adding shaking to the traditional white tea process; **YRB**: sample made by adding shaking and rolling in the traditional white tea processing; **YLRB**: sample made by adding shaking, rolling, and freezing to the traditional white tea process.

Sample preparation for optimal processing technique investigation: The experimental design incorporated four distinct processing methods. The control group (CK) followed the traditional white tea processing technique (fresh leaves; withering; drying; final product). The experimental groups included YB (withering with the shaking process), YRB (moderate withering with the shaking and rolling processes), and YLRB (moderate withering with the shaking, freezing, and rolling processes). Withering conditions were strictly controlled at 40 °C with 65% relative humidity, utilizing a 68 cm three-blade industrial fan operating at 1400 r/min for air circulation, continuing until the moisture content reached 52%. The shaking process was conducted in a drum shaker at 10 r/min for 10 min. Post-shaking, samples were immediately packaged and frozen at −20 °C for 5 h, followed by clump disintegration and cold-air cooling. The rolling process was executed at a 25% pressure intensity (achieved by compressing the roller cover to one-quarter of the barrel height with a consistent leaf quantity) for 9 min. Final processing involved low-temperature drying at 75 °C for 2 h, reducing the moisture content to below 5% to obtain the finished tea samples (Figure 1). For subsequent analysis, the tea samples were processed according to GB/T 8303-2013 “Preparation of Ground Tea Samples and Determination of Dry Matter Content”. The samples were ground, sieved through a 30-mesh screen, and stored at −20 °C for future experimental use.

### 2.3. Sensory Evaluation

Eight professional tea evaluators were selected to form a sensory evaluation panel. The sensory assessment was conducted in strict accordance with the national standard “Methodology for Sensory Evaluation of Tea” (GB/T 23776-2018), with each sample evaluated in triplicate.

Quantitative descriptive analysis (QDA) has been established as an effective methodology for sensory evaluation, particularly in assessing the aroma and flavor profiles of tea products [17,18]. In this study, following the guidelines of GB/T 16861-1997 “Sensory Analysis by Multivariate Analysis Method for Identification and Selection of Descriptors Used to Establish Sensory Profiles” and incorporating modifications based on the methodology developed by Hao et al. [19], a panel of 10 professional tea evaluators (comprising 5 male and 5 female members) conducted sensory evaluations in accordance with GB/T 23776-2018 “Methodology for Sensory Evaluation of Tea”. The sensory evaluation process involved assessing the aroma and flavor characteristics of the tea samples using a standardized 0–5-point scale, where the numerical values corresponded to the intensity levels of specific sensory attributes. Prior to the evaluation sessions, all panel members received comprehensive training and provided informed consent for their participation in the experimental procedures.

### 2.4. Determination of Macro-Compositions of Tea

The total content of tea polyphenols was determined following the Chinese National Standard GB/T 8313-2018 “Determination of Tea Polyphenols and Catechins Content in Tea” [20]. The analysis was performed using gallic acid as the standard reference, with absorbance measurements taken at a 765 nm wavelength for the quantitative determination of the tea polyphenol content.

The free amino acid content was quantified according to the ninhydrin colorimetric method specified in GB/T 8314-2013 [21].

The soluble sugar content was determined using the anthrone–sulfuric acid colorimetric method [22]. For this analysis, 1 mL of tea extract was mixed with 4 mL of distilled water, from which a 0.5 mL aliquot was combined with 4.0 mL of anthrone–sulfuric acid reagent in an ice bath. The mixture was then heated in a boiling water bath for precisely 7 min, followed by absorbance measurement at 620 nm.

The total caffeine content in the tea samples was determined using the ultraviolet spectrophotometric method, as specified in the Chinese National Standard GB/T 8312-2013.

The analysis of catechin and theaflavin components was conducted following the Chinese National Standard GB/T 8313-2018 with necessary modifications. The extraction procedure involved combining 0.3 g of tea powder with 5 mL of preheated 70% methanol (70 °C) in a 10 mL centrifuge tube. The mixture was thoroughly vortexed and subsequently incubated in a 70 °C water bath for 15 min, with stirring every 5 min. Following cooling to ambient temperature, centrifugation was performed at 4000 rpm for 8 min. The supernatant was transferred to a new 10 mL centrifuge tube. This extraction process was repeated twice, and the collected supernatants were pooled. The combined extract was then adjusted to a final volume of 10 mL using distilled water. For a chromatographic analysis, a 2 mL aliquot of the sample extract was diluted to 10 mL with ultrapure water and filtered through a 0.45 μm organic membrane prior to injection. The HPLC analysis was performed under the following conditions: Ascentis^®^ RP-Amide column (250 mm × 4.6 mm, 5 μm); mobile phase A consisting of glacial acetic acid–water (500:1, *v*/*v*); mobile phase B consisting of 100% acetonitrile; flow rate maintained at 1 mL/min; column temperature set to 35 °C; and a gradient elution program spanning 0–15 min.

The amino acid composition was determined according to the method of Yi-Wen Miao et al. [3], with appropriate modifications as follows: For sample preparation, 3 g of tea powder was weighed into a 500 mL Erlenmeyer flask. Then, 450 mL of boiling distilled water was added, and the mixture was immediately subjected to a boiling water bath. After immersion for 50 min, it was extracted and filtered. The filtrate was transferred to a 500 mL volumetric flask, and the volume was adjusted to 500 mL with cooled distilled water. The solution was shaken well and set aside. After the tea extract was prepared, 800 μL of tea extract, 400 μL of NFB, and 400 μL of Na_2_CO_3_-NaHCO_3_ buffer (pH = 9.2) were accurately pipetted into a 5 mL centrifuge tube. The mixture was stirred well and then placed in a water bath at 60 °C for a dark reaction for 60 min. After the reaction, the solution was removed and cooled to room temperature. Subsequently, 1600 μL of KH_2_PO_4_-NaOH buffer (pH = 7.0) was added, and the mixture was vortexed for 2 min. The reaction was kept in the dark at room temperature for 18 min. Finally, 1 mL of the reaction solution was aspirated through a 0.22 μm membrane. For chromatography, an Agilent EXTEND C18 column (4.6 × 250 mm, 5 μm) was used, with a detection wavelength of 360 nm.

### 2.5. Determination of Volatile Components of Tea

The volatile compounds were analyzed by GC-MS using a modified method based on the original protocol of Chengzhe Zhou [23]. A crushed tea sample weighing 0.5000 g (sieved through a 30-mesh sieve), 2.5 mL of saturated NaCl solution, and 3 μL of 4-tert-butylcyclohexanol (30 mg/L) solution were placed in a headspace vial and sealed with a cap. Headspace conditions: oven temperature: 80 °C; sampling needle temperature: 100 °C; transfer line temperature: 120 °C; sample equilibration time: 30 min; trap temperature: Hi 280 °C, Lo 40 °C (hold for 5 min, dry blow for 1 min, and resolve for 0.5 min); column pressure: 12 psi; resolving pressure: 12 psi; and headspace outlet shunt. Gas chromatography (GC) conditions: column: Elite-5MS capillary column (30 m × 0.25 mm × 0.25 μm); inlet temperature: 250 °C; carrier gas: helium (purity > 99.999%); and heating program: initial column temperature 40 °C, held for 3.5 min; increased to 100 °C at 8 °C/min and held for 4 min; then raised to 180 °C at 7 °C/min and held for 5 min; finally increased to 230 °C at 12 °C/min and held for 5 min. The total running time was 46 min.

### 2.6. Statistical Analysis

The data were processed using Excel. Statistical analysis was performed using Statistical Package for the Social Sciences 25 (SPSS 25) to determine the significance of differences (Waller–Duncan method, *p* < 0.05). Double Y-axis scatter plots, while bar plots, and radar plots were generated using Origin Pro 2024. Cluster heat maps were constructed using TBtools v1.0987663. Multivariate statistical analysis was conducted using SIMCA 14.1.

## 3. Results and Discussion

### 3.1. Results of the Study of Optimal Machining Process Parameters

#### 3.1.1. Influence of Different Shaking Speeds on the Quality of Summer Tea

The results of the sensory evaluation indicated that the shaking speed had a significant impact on the quality of summer tea (Table S2). As the shaking speed gradually increased, the color of the dry tea darkened, the uniformity of the leaves showed a trend of initial increase followed by a decrease, the color of the tea broth shifted from yellow and bright to orange and bright, the aroma evolved from grassy to floral and fruity, the taste became fresher and sweeter, the bud tip at the base of the leaf gradually disappeared, the breakage of the leaf fragments increased, and the brightness of the leaf base color increased and then decreased. The sensory scores demonstrated an upward trend, followed by a subsequent downward trend. The sensory quality of summer tea exhibited an enhancement from a shaking speed of 4 to 10 r/min, and the sensory scores achieved a maximum value of 92.33 at a shaking speed of 10 r/min. The shaking process has been shown to promote the formation of volatile compounds, including dimethyl sulfide, 2-methylbutanal, and linalool [24,25]. These compounds have been found to effectively reduce the grassy flavor and enhance the sweetness of tea infusion. This finding is consistent with the experimental results obtained in this study. The sensory evaluation results suggest that moderately increasing the shaking speed could effectively enhance the appearance, aroma, taste, and leaf quality of summer tea, with the optimal sensory quality achieved at a shaking speed of 10 r/min.

**Figure 2:**
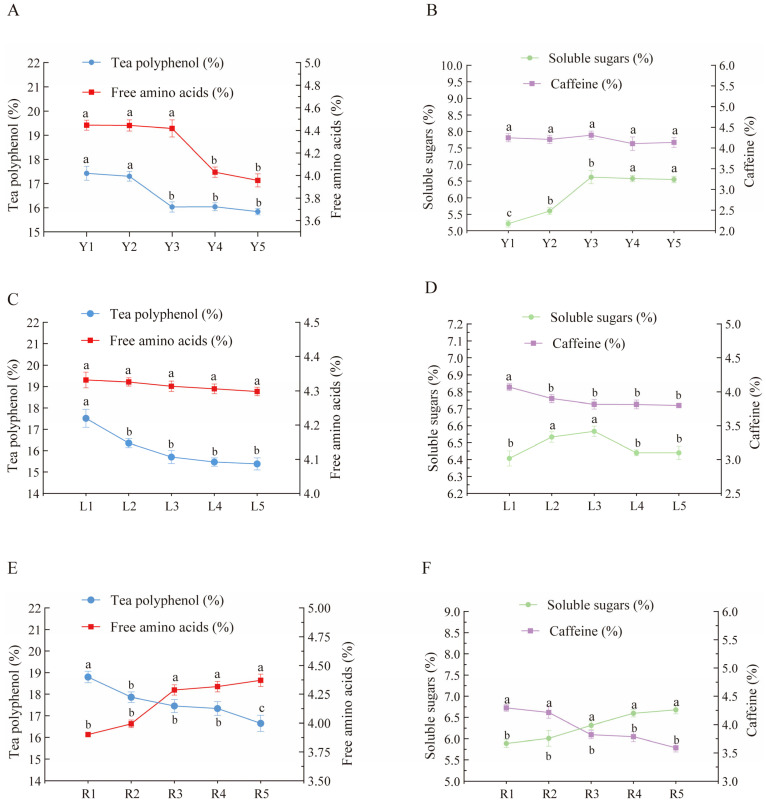
(**A**) Effect of shaking speed on the polyphenols and free amino acids of summer tea. (**B**) Effect of shaking speed on soluble sugars and caffeine of summer tea. (**C**) Effect of freezing time on polyphenols and free amino acids of summer tea. (**D**) Effect of freezing time on soluble sugars and caffeine of summer tea. (**E**) Effect of rolling time on polyphenols and free amino acids of summer tea. (**F**) Effect of rolling time on soluble sugars and caffeine of summer tea. Note: The letters “abc” are used to indicate significant differences between treatments (*p* < 0.05).

An analysis of the physicochemical compositions of summer tea under varying shaking speeds (Figure 2A,B) revealed that the contents of tea polyphenols and free amino acids decreased as the shaking speed increased. When the shaking speed exceeded 7 rpm, the contents of tea polyphenols decreased significantly (*p* < 0.05), and then the decrease became smaller. When the shaking speed exceeded 10 rpm, the content of free amino acids decreased significantly (*p* < 0.05). In contrast, the soluble sugar content gradually increased with the increase in the shaking speed, and its maximum value reached 6.62%, but when the shaking speed exceeded 10 r/min, the change in the soluble sugar content tended to level off. The caffeine content showed an undulating change with the increase in the shaking speed. The reasons for the changes in these substances might be that the shaking process promotes the opening of stomata and disrupts the cell structure, regulates the activity of polyphenol oxidase, accelerates the degradation of proteins and polyphenols, and promotes the hydrolysis of glycoside compounds, thereby reducing the contents of tea polyphenols and amino acids in tea, promoting the generation of soluble sugars, and improving the quality of tea [26,27]. This result agrees with the sensory evaluation. Taken together, the optimum shaking speed of 10 r/min for summer tea can be preliminarily determined from the single-factor experiment.

#### 3.1.2. Influence of Different Freezing Times on the Quality of Summer Tea

The findings of the sensory analysis indicated that the freezing time had a significant impact on the quality of summer tea (Table S3). As the freezing time lengthened, the appearance of the dry tea changed from deep green with leaf trichomes to dark green with leaf trichomes and then to dark yellow with fewer leaf trichomes. The color of the tea broth gradually deepened; the aroma gradually enriched from slightly floral to distinctly fruity and floral; the taste changed from fresh, mellow, and astringent to fresh, mellow, and sweet and then to slightly fresh and astringent; the bitterness and astringency first decreased and then increased; and the brightness of the bottom of the leaf gradually decreased. The sensory quality of the summer tea increased with the freezing time from 2 h to 4 h, and the sensory scores of the summer tea achieved the maximum value (87.19) at a freezing time of 4 h. Freezing enhances sensory preferences, aligning with prior research findings [12]. The results of the sensory evaluation show that a moderate extension of the freezing time was beneficial to the development of the appearance, color, aroma, and taste qualities of summer tea, but it had a negative effect on the quality of the leaf base, and the quality of summer tea treated with a freezing time of 4 h was the best.

A comparison of the physicochemical compositions of summer tea subjected to different freezing time treatments revealed a gradual decrease in the levels of tea polyphenols, free amino acids, and caffeine with an increase in the freezing time (Figure 2C,D). This decline was particularly pronounced when the freezing time exceeded 2 h. Conversely, the soluble sugar content exhibited a tendency to increase and then decrease with the prolongation of the freezing time, reaching a maximum of 6.56% at 6 h. The permeability of the cell membrane was increased by freezing, and the substances in the cell after thawing moved from the intracellular to the extracellular space, causing the oxidation of polyphenols [12]. Niu Xiaojun et al. [28] found that the contents of tea polyphenols and caffeine in fresh leaves decreased gradually with increasing freezing time when compared to those in unfrozen fresh leaves. The results obtained from this study were consistent with the sensory evaluation. The optimal freezing time for summer tea was initially determined to be 4 h through the single-factor experiment.

#### 3.1.3. The Influence of Different Rolling Times on the Quality of Summer Tea

The findings of the sensory analysis indicated that the rolling time had a significant impact on the quality of summer tea (Table S4). As the rolling time gradually increased, the color of the dry tea changed from dark green to yellowish brown; the firmness increased; the color of the tea liquor changed from orange-yellow and bright to orange-red and brighter and then to brownish-red and dark; the quality of the tea liquor decreased; the aroma was gradually enriched from a slightly floral aroma to a distinctly fruity and floral aroma; the taste of the tea broth changed from fresh and bright, bitter, and astringent to fresh and bright, mellow, and sweet and then to soft and astringent; the bud tip at the base of the leaf gradually disappeared, appearing broken; and the brightness increased and then decreased. The sensory scores demonstrated a general upward trend, followed by a subsequent downward trend, and the maximum sensory score (92.72) was attained at a rolling time of 9 min. This effect occurred because rolling accelerates polyphenol oxidation while enhancing β-glucosidase activity to release glycoside-derived volatiles, which primarily exist as glycosides imparting floral, honey, and fruity flavor notes [29]. The results of the sensory evaluation show that a moderate rolling time could effectively improve the appearance, aroma, taste, and leaf bottom of summer tea, but it was unfavorable to the quality formation of the soup color, and the quality of summer tea treated with a rolling time of 9 min was the best.

A comparison of the physicochemical compositions of summer tea under different rolling time treatments revealed a gradual decrease in the contents of tea polyphenols and caffeine with an increase in the rolling time (Figure 2E,F). Conversely, the levels of free amino acids and soluble sugars exhibited a gradual increase with an increase in the rolling time. However, when the rolling time exceeded 6 min, there was a significant increase in the levels of free amino acids and soluble sugars, but the rise decreased after 9 min. During the rolling process, the cells are ruptured, and the contents penetrate to the surface of the leaves and undergo a variety of physical changes [30]. The results of this study are consistent with those in a report by Dong Chen et al. [31] on the influence of rolling time on the quality of green tea. The optimal twisting time for summer tea was initially determined through a one-way test, which revealed that the ideal time was nine minutes.

#### 3.1.4. Orthogonal Experiment on the Optimal Processing Technology of Summer Tea

Three factors—shaking speed (A), freezing time (B), and rolling time (C)—were selected for an orthogonal test of L_9_(3^4^). The sensory scores were used as the indexes of the investigation to ascertain which factors had a significant effect on the test results and to determine the optimal technological conditions for summer tea. The outcomes of the orthogonal test are presented in Table 1. The table indicates that the order of influence of the three factors on the sensory quality of summer tea obtained from the new process is A (shaking speed) > B (freezing time) > C (rolling time). Polar analysis results indicated that the optimal combination of summer tea processing technology is A2B3C2, the optimal shaking speed is 10 r/min, the optimal freezing time is 5 h, and the optimal rolling time is 9 min.

To further verify the accuracy of the results, the results of the orthogonal tests were analyzed by ANOVA, which reflects the significance of the effects of experimental factors on the overall sensory quality of summer tea [30]. As shown in Table S5, the mean squares of the shaking speed, freezing time, and rolling time were all larger than the mean squares of the errors of the null column, indicating that the effects of these three factors on sensory quality reached a significant level (*p* < 0.05), which was consistent with the results of the analysis of variance, so the shaking speed, freezing time, and rolling time had a great influence on the overall sensory quality of the summer tea. Parallel verification experiments were conducted to validate the optimal process parameters derived from orthogonal experiments (Table S6). The results show that the summer tea produced under the conditions of a 10 r/min shaking speed, 5 h freezing time, and 9 min rolling time yielded sensory evaluation scores of 90.16, 90.95, and 90.37, with an average score of 90.49—higher than the maximum score (89.65) obtained in the orthogonal experiments. These findings demonstrate the accuracy and reproducibility of the method, confirming these parameters as the optimal processing conditions for the novel summer tea production technique.

### 3.2. Results of the Optimal Machining Process Study

#### 3.2.1. Quantitative Descriptive Analysis

**Figure 3:**
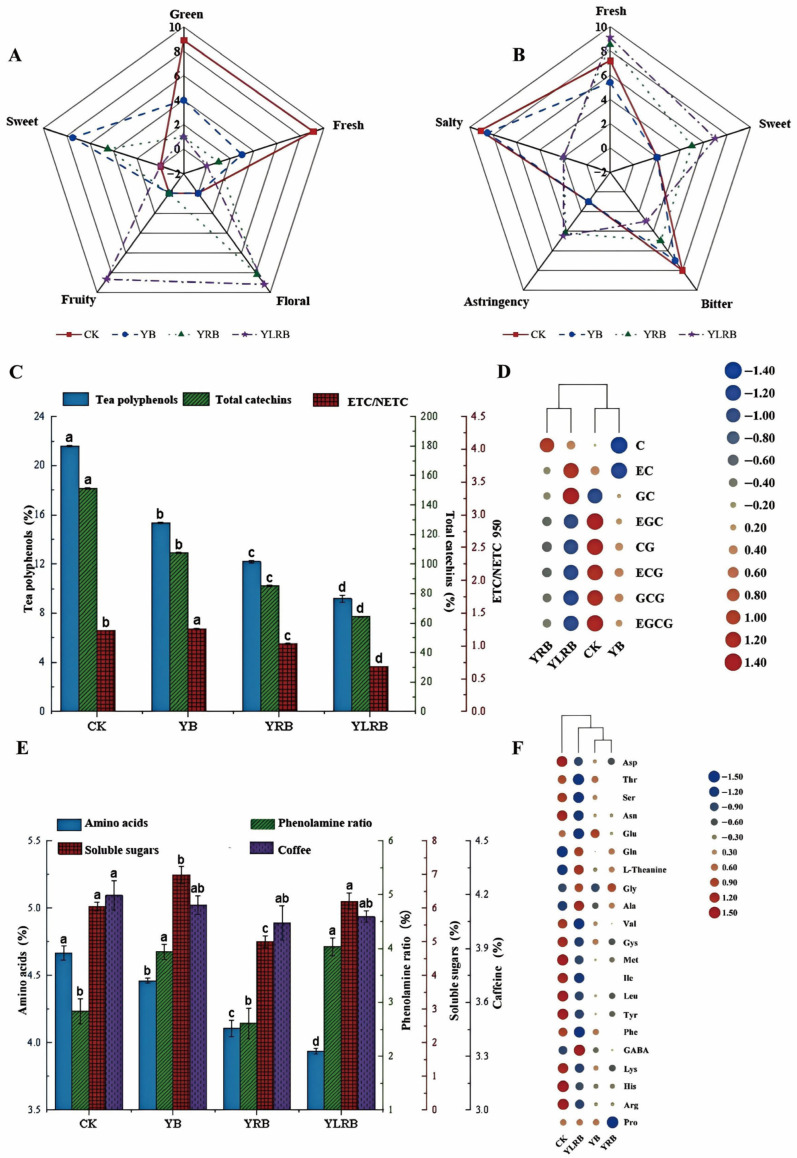
(**A**) Radar chart of the sensory evaluation of the aroma of summer tea produced by different processing methods. (**B**) Radar chart of the sensory evaluation of summer tea flavor made by different processing methods. (**C**) Changes in polyphenols, total catechins, and the ratio of ester and non-ester catechins of summer tea produced by different processing methods. (**D**) Heat map of catechin content in summer tea produced by different processing methods. (**E**) Ratio of amino acid composition in summer tea made by different processing methods. (**F**) Heat map of amino acid content in summer tea made by different processing methods. Note: The letters “abcd” are used to indicate significant differences between treatments (*p* < 0.05).

The organoleptic characteristics of summer teas produced by different processing methods are shown in Figure 3A,B. The control (CK) sample has a grassy and fresh aroma, a strong bitter and salty taste, and a certain freshness. The YB sample has an obvious sweet and partly grassy aroma, with an obvious bitter and salty taste. The YRB sample has a floral aroma and a persistent sweet aroma but an obvious astringent taste. The YLRB sample has a long-lasting floral and fruity aroma, accompanied by a distinctive freshness and sweetness in taste. These results indicate that the summer tea produced under the YLRB process exhibits optimal organoleptic quality.

#### 3.2.2. Influence of Processing Technology on Catechin in Summer Tea

Catechins are the main components of tea polyphenols. From CK to YB, then to YRB, and then to YLRB, with the development of the processing methods, the content of total catechins gradually decreased. Compared with the control group, the total catechin content decreased by 28.95%, 43.57%, and 57.42%, respectively. A total of eight catechin monomers were detected in all tea samples. Cluster analysis grouped CK and YB together and YRB and YLRB together. The contents of EGC, CG, ECG, GCG, and EGCG were significantly higher in CK and YB than in YRB and YLRB (*p* < 0.05). The eight catechins were classified into four non-ester catechins (C, EC, GC, and EGC) and four ester catechins (CG, ECG, GCG, and EGCG) [32]. It was observed that, as the processing method transitioned from CK to YB to YRB to YLRB, there was a gradual increase in the percentage of non-ester catechin monomers, accompanied by a simultaneous decrease in the percentage of ester catechin monomers (Figure 3C,D). This change was consistent with the reduction in bitterness and astringency in the sensory evaluation. Xiaofeng Lu et al. [33] also showed that shaking promoted the conversion of EGCG to GA, ECG, EC, and C and reduced the bitterness and astringency of tea broth. Freezing decreased the activity of polyphenol oxidase; however, the combination of shaking and freezing treatments resulted in increased levels of GC, C, and CG [13]. During the rolling process, there is a large loss of cellular water, the cell structure is damaged, and it is easily combined with oxygen for an oxidation reaction, resulting in a gradual decrease in the catechin content [15]. In summary, the findings of this study demonstrate that the methods of YLRB have a significant impact on the content of tea polyphenols in summer tea.

#### 3.2.3. Influence of Processing on Total Tea Polyphenol, Amino Acid, Phenol–Ammonia Ratio, Soluble Sugar, and Caffeine Contents in Summer Tea

A physicochemical examination was conducted to determine the total tea polyphenol, amino acid, phenol–ammonia ratio, soluble sugar, and caffeine contents in summer tea with different processing techniques (Figure 3E). Tea polyphenols are important substances that affect the flavor intensity and astringency of tea infusion. Amino acids are the main internal substances that affect the freshness of tea broth. The contents of tea polyphenols and amino acids in the YB, YRB, and YLRB tea samples were significantly lower than in the CK treatment (*p* < 0.05). The phenol–ammonia ratio showed a strong correlation with the tea broth taste harmony. Compared to CK, this ratio remained relatively stable in the YRB treatment but increased significantly (*p* < 0.05) in both the YB and YLRB treatments. Soluble sugar, the predominant sweet-tasting compound in tea broth, significantly increased (*p* < 0.05) in YB, significantly decreased (*p* < 0.05) in YRB, and showed no significant difference (*p* > 0.05) in YLRB compared to the control. Caffeine was identified as the primary bitter and astringent flavor constituent in the tea broth, and no statistically significant differences (*p* > 0.05) in the caffeine content were observed among the summer teas produced by the four distinct processing methods. Changes in these inclusions may result from freezing-induced cell structure disruption, while shaking accelerates amino acid decarboxylation and protein hydrolysis, and rolling enhances polyphenol oxidase and other enzyme activities [13,14,34].

#### 3.2.4. Influence of Processing on the Amino Acid Composition of Summer Tea

The free amino acids in tea serve as key components responsible for determining its fresh flavor and aroma characteristics [35]. A total of 21 amino acids were identified across all tea samples (Figure 3F and Table S7). These amino acids were categorized into three groups based on their taste characteristics: fresh (Asp, Asn, Glu, and Gln), sweet (Thr, Ser, Gly, Ala, Cys, Met, and Pro), and bitter (Val, Ile, Leu, Tyr, Phe, Lys, His, and Arg). The transition of tea processing from CK to YB, YRB, and YLRB resulted in a substantial alteration in the composition of the amino acid content in the tea. There was a gradual increase in the proportion of sweet and fresh amino acids, accompanied by a gradual decrease in the proportion of bitter amino acids. In comparison with the control group (CK), the levels of fresh and sweet amino acids in the YLRB treatment group exhibited an increase of 4.96% and 2.95%, respectively. Concurrently, the levels of two specific amino acids, GABA and L-theanine (a distinctive tea component [36,37]), increased. As indicated by the results of the study, the GABA content exhibited an increase of 0.51%, while the L-theanine content demonstrated a more substantial increase of 5.77%. Conversely, the content of bitter amino acids exhibited a decrease of 2.15%. This outcome indicates that the enhancement of processing not only elevated the flavor profile of the tea but also augmented the concentration of its functional components. Shaking, freezing, and rolling treatments all disrupt cellular structures and accelerate enzymatic reactions (e.g., deamination and decarboxylation), thereby increasing the levels of amino acids such as phenylalanine, GABA, and tyrosine; these processes further promote the oxidative polymerization of tea polyphenols and amino acids, ultimately reducing the content of bitter amino acids [13,15,38]. Cluster analysis classified the four summer tea samples into two distinct groups, with CK forming one separate cluster and the other three samples (YB, YRB, and YLRB) grouped together. The CK sample exhibited significantly higher contents of Asp, Asn, Met, Ile, Leu, and Tyr than YB, YRB, and YLRB. In conclusion, proper shaking, rolling, and freezing can reduce the content of bitter amino acids and improve the quality of tea leaves.

#### 3.2.5. Influence of Processing Technology on the Volatile Compounds in Summer Tea

**Figure 4:**
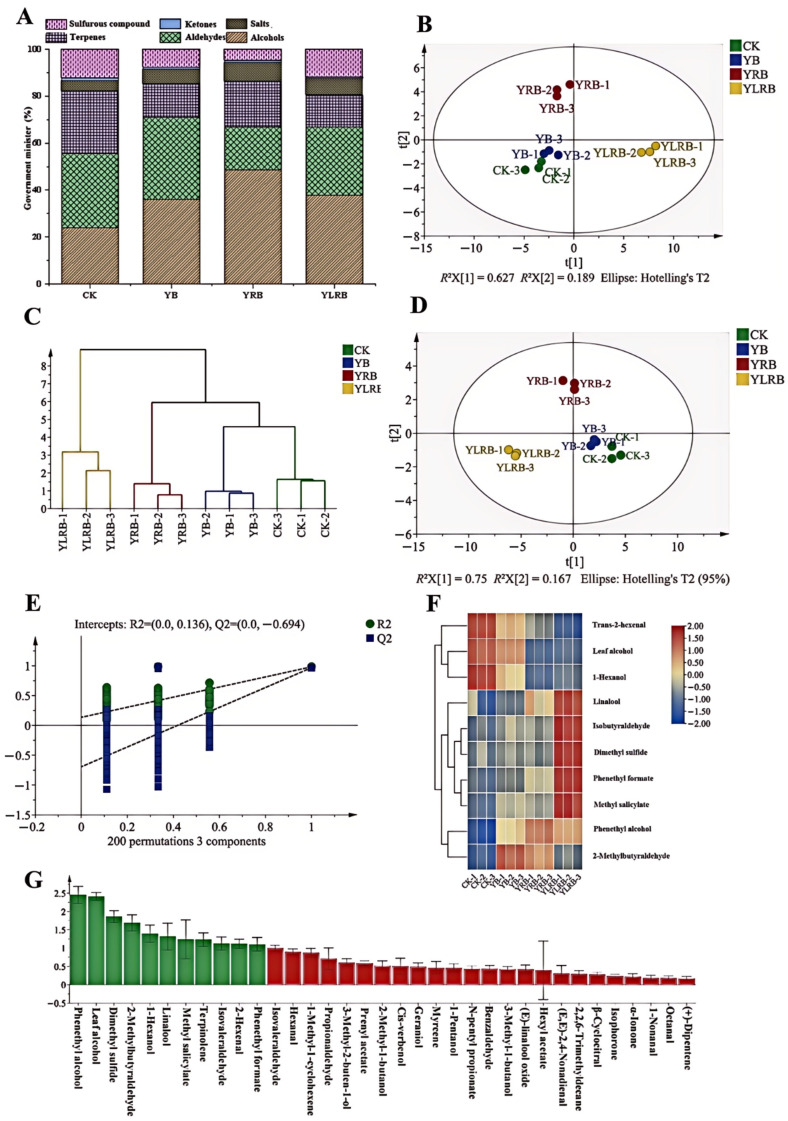
(**A**) Aroma composition of summer tea made by different processing methods. (**B**) Scatter plot of principal component analysis (PCA) of summer tea made by different processing methods. (**C**) Hierarchical cluster analysis (HCA) of summer tea produced by different processing methods. (**D**) Scatter plot of orthogonal partial least squares-discriminant analysis (OPLS-DA) of summer tea made by different processing methods. (**E**) OPLS-DA replacement test of summer tea made by different processing methods. (**F**) Thermogram of key volatile components of summer tea made by different processing methods. (**G**) Variable importance in projection scores of the OPLS-DA model of the aroma components of summer tea made by different processing methods.

In this study, GC-MS was utilized to analyze the aroma components of four summer teas with divergent processing techniques. A total of 35 volatile compounds, encompassing aldehydes (10), terpenes (10), alcohols (7), esters (5), ketones (2), and sulfur-containing compounds (1), were identified (Figure 4A and Table S8). The highest content of total aroma substances was found in the YLRB sample (35.96 μg/g), which was significantly higher than that in the CK sample (21.11 μg/g) (*p* < 0.05). The aroma compounds primarily consisted of higher concentrations of alcohols (25.23–55.05%) and aldehydes (22.02–33.18%). Alcohols typically impart floral and fruity notes, while aldehydes generally exhibit grassy, floral, and fruity aromas [39]. It is noteworthy that myrcene and D-limonene—volatile compounds characterized by floral and fruity aromas—are signature components of floral black tea [40]. But these compounds were detected exclusively in the CK and YLRB samples. As the processing method evolved from CK to YB, YRB, and YLRB, there was a gradual decrease in compounds with a grassy aroma, accompanied by a concurrent increase in compounds with floral and fruity aromas. This is in alignment with the outcomes of the sensory review.

To investigate the aroma composition differences in summer teas processed through different techniques, this study employed principal component analysis (PCA) (Figure 4A), hierarchical cluster analysis (HCA) (Figure 4C), and orthogonal partial least squares-discriminant analysis (Figure 4D) to analyze 35 aroma compounds across the four tea types. Both PCA and HCA revealed clear separation of the four tea groups, demonstrating significant differences in their aroma profiles. The orthogonal partial least squares-discriminant analysis (OPLS-DA) model further enabled distance-based similarity assessment, where a closer sample proximity indicated higher aromatic similarity, and vice versa [34]. The OPLS-DA model’s reliability was confirmed through 200 permutation tests in cross-validation (Figure 4E). The analytical results showed pronounced differentiation between the YRB and YLRB samples, while YB and CK exhibited relatively minor compositional differences.

#### 3.2.6. Main Aroma Compounds of Summer Tea Made by Different Processing Techniques

The rOAV (relative odor activity value), as a comprehensive evaluation method, determines the aroma contribution of individual components by integrating both their concentrations and odor thresholds [41,42]. In tea aroma analysis, compounds with rOAV > 1 are generally recognized as contributors to aroma profile formation, while those with rOAV > 10 play a dominant role in determining aroma characteristics [43,44]. The rOAV analysis identified 28 aroma compounds (rOAV > 1) in the four summer tea samples (Table S9). Among these, CK, YB, YRB, and YLRB contained 24, 23, 25, and 27 compounds (rOAV > 1), respectively, with YLRB exhibiting the highest number. Notably, the number of high-impact components (rOAV > 10) showed progressive increases: 13 in CK, 16 in YB, and 17 in both YRB and YLRB. Among these, 17 key aroma substances—including linalool, phenethyl formate, methyl salicylate, isobutyraldehyde, and dimethyl sulfide—were particularly significant in YLRB. These components collectively contribute to YLRB’s distinctive and persistent floral–fruity aroma profile [45].

#### 3.2.7. Analysis of the Main Volatile Compounds of Summer Tea in Different Processing Techniques

Variable importance in projection (VIP) can be used to quantitatively assess the contribution of each variable to classification in OPLS-DA models. Variables with VIP values > 1 are generally considered to significantly contribute to classification [46]. In the four summer tea samples, 11 aroma substances exhibited VIP values exceeding 1 (Figure 4G). Based on the dual criteria of odor activity value rOAV > 1 and VIP > 1, 10 major differential volatile compounds were identified: phenylethanol, leaf alcohol, dimethyl sulfide, 2-methylbutyraldehyde, 1-hexanol, linalool, methyl salicylate, isobutyraldehyde, 2-hexenal, and phenethyl formate. Cluster analysis based on the relative contents of these 10 components revealed distinct compositional patterns (Figure 4F). Three characteristic volatile compounds were identified in the CK samples: leaf alcohol (grassy aroma), 1-hexanol (grassy aroma), and 2-hexenal (fresh aroma). The YB sample was characterized by 2-methylbutyraldehyde, presenting a primarily sweet aroma with secondary grassy notes. Phenylethanol (rosy aroma) emerged as the characteristic component in the YRB samples. The YLRB sample contained five characteristic components, with linalool, phenethyl formate, and methyl salicylate contributing predominantly fruity notes complemented by floral undertones [47]. Dimethyl sulfur has a cooked corn-like aroma and is a key aroma substance in fresh green teas such as Dragon Well [25,48]. Isobutyraldehyde can be used as a food flavor to configure various fruit flavors. Leaf shaking promotes the degradation of fatty acids, which, in turn, promotes the conversion of alcohol to esters, resulting in a lower content of alcohol with a grassy flavor [49]. The combination of freezing and shaking has been shown to be a more effective method of destroying the cell structure, enhancing the activity of enzymes, and increasing the hydrolysis of glycoside aroma substances. This process has been found to enrich the content of aroma substances [13]. The process of rolling also increases enzyme activity and promotes the formation of floral and fruity compounds [50]. Overall, freezing, shaking, and rolling processes can improve the quality of summer tea.

## 4. Conclusions

In this study, we determined that the best processing parameters for improving summer tea quality were fresh leaves, room-temperature cold-air withering for 6.5 h, shaking at 10 rpm for 10 min, −20 °C freezing for 5 h, 25% strength rolling for 9 min, and drying at 75 °C for 2 h. This process was shown to effectively reduce the relative content of ester catechins; increase the contents of fresh and sweet amino acids, GABA, and L-theanine; and reduce the content of bitter amino acids. Consequently, the bitterness and astringency of the summer tea were reduced, thereby improving its quality. According to GC-MS analysis, the summer tea produced by optimal processing exhibited five distinctive volatile compounds, namely, methyl salicylate, phenethyl formate, linalool, dimethyl sulfide, and isobutyraldehyde. The volatile profiles were dominated by floral and fruity flavors, except for dimethyl sulfide, which exhibited pronounced aroma characteristics of cooked corn. These findings provide a foundation for enhancing the quality of summer tea. However, differences in key metabolites during the shaking, freezing, and rolling processing of summer tea need to be analyzed accurately and quantitatively.

## Figures and Tables

**Table 1:** Results of the orthogonal test on processing parameters of summer tea.

No.	A: Rotation Speed (r/min)	B: Freezing Time (h)	C: Rolling Time (min)	D (Blank Column)	Sensory Evaluation (Point)
1	1	1	1	1	76.45
2	1	2	2	2	83.46
3	1	3	3	3	84.65
4	2	1	2	3	84.32
5	2	2	3	1	89.65
6	2	3	1	2	87.82
7	3	1	3	2	75.64
8	3	2	1	3	78.32
9	3	3	2	1	82.42
K1	244.56	236.41	242.59	248.52	
K2	261.79	251.43	250.20	246.92	
K3	236.38	254.89	249.94	247.29	
R	8.47	6.16	2.54	0.53	
Prioritize Factors	1	2	3	4	
Optimal Solution			A2B3C2		

Note: K1 represents the sum of all sensory evaluation scores at level 1 of this factor; K2 represents the sum of all sensory evaluation scores at level 2 of this factor; K3 represents the sum of all sensory evaluation scores at level 3 of this factor; R represents the extreme difference of the K value of the corresponding factor at different levels.

## Data Availability

The data presented in this study are available on request from the corresponding author.

## Supplementary Materials

The following supporting information can be downloaded at: https://www.mdpi.com/article/10.3390/foods14183159/s1, Table S1: New technology for summer tea, optimizing the orthogonal test factors and levels; Table S2: Sensory analysis of summer tea with different shaking rotation speeds; Table S3: Sensory analysis of summer tea with different freezing times; Table S4: Sensory analysis of summer tea with different rolling times; Table S5: Orthogonal test analysis of ANOVA; Table S6: Results of the validation test; Table S7: Analysis of amino acid fractions in summer tea produced by different processing techniques; Table S8: Volatile compound composition of summer tea processed with different techniques; Table S9: The rOAV values, aroma characteristics, and thresholds of volatile compounds in summer tea produced by different processing techniques.
